# Rapavir, a novel inhibitor of sodium taurocholate cotransporting polypeptide, potently blocks hepatitis B virus entry

**DOI:** 10.1038/s41392-025-02214-x

**Published:** 2025-04-23

**Authors:** Jia He, Haibo Yu, Kunling Song, Ailong Huang, Yongjun Dang, Juan Chen, Zufeng Guo

**Affiliations:** 1 Basic Medicine Research and Innovation Center for Novel Target and Therapeutic Intervention (Ministry of Education), College of Pharmacy & Department of Breast and Thyroid Surgery of the Second Affiliated Hospital, Chongqing Medical University, Chongqing, China; 2 Institute for Viral Hepatitis, Key Laboratory of Molecular Biology for Infectious Diseases (Ministry of Education), Department of Infectious Diseases, The Second Affiliated Hospital, Chongqing Medical University, Chongqing, China; 3 College of Biomedical Engineering, Chongqing Medical University, Chongqing, China

**Keywords:** Drug screening, Chemical biology

**Dear Editor**,

Chronic hepatitis B virus (HBV) is a global health problem closely associated with a spectrum of liver diseases. Current clinical treatment options for HBV infection are generally not curative, highlighting the need for the development of novel therapeutics. Sodium taurocholate cotransporting polypeptide (NTCP) was identified as a functional receptor for HBV entry, making it a promising therapeutic target for developing novel anti-HBV agents. Although considerable efforts have been made to develop small molecule inhibitors against NTCP, many of these compounds suffer from low inhibitory potency and lack of efficacy in vivo.^1^ Therefore, the development of novel NTCP inhibitors with high specificity and efficacy is of great importance.

Inspired by the natural products rapamycin and FK506, we recently generated a macrocycle library called rapafucins.^2^ Through high-throughput screenings, we identified several promising inhibitors against diverse targets, suggesting that rapafucins are versatile chemical probes and drug leads.^2,3^ To identify novel inhibitors of NTCP, we screened a rapafucin 3D microarray containing 3918 individual rapafucins^3^ and found 15 potential hits (Fig. 1a). Among those hits, rapafucin HP07-C6 exhibited the highest inhibitory activities against both taurocholic acid-d_4_ (TCA-d_4_) uptake and HBV infection with IC_50_ values of 79 ± 8.2 nM (TCA-d_4_), 443 ± 19.8 nM (HBeAg) and 390 ± 6.2 nM (HBV 3.5-kb RNA), respectively (Fig. 1a). To further improve the potency of HP07-C6, we conducted a structure-activity relationship (SAR) study by altering the tetrapeptide effector domain of HP07-C6. Among 71 analogs synthesized and tested, JH-B10 showed significantly improved inhibitory activity against both TCA-d_4_ uptake and HBV infection with IC_50_ values of 1.8 ± 0.3 nM (TCA-d_4_), 63 ± 7.2 nM (HBeAg), and 22 ± 2.3 nM (HBV 3.5-kb RNA), respectively (Fig. 1a). Given its potent inhibitory effect on HBV infection, we named JH-B10 rapavir.

**Fig. 1:**
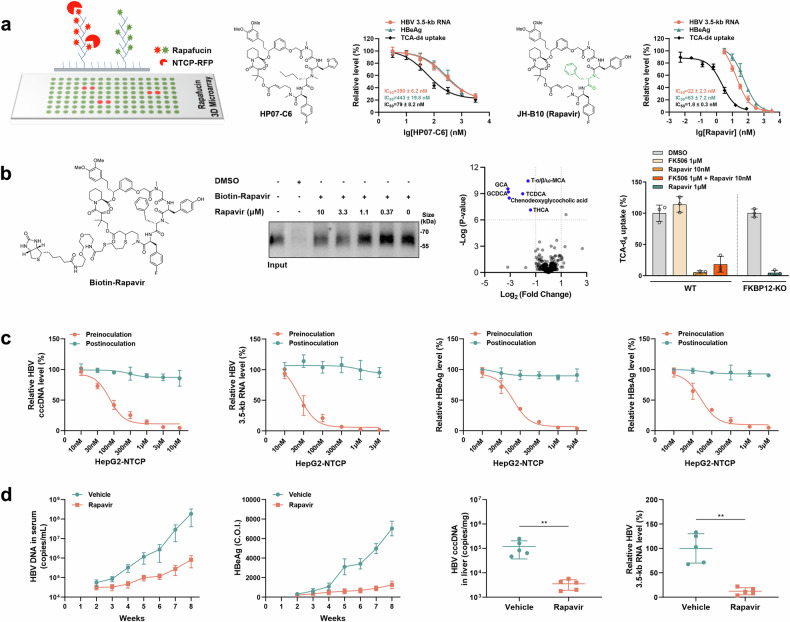
Discovery of rapavir as a novel inhibitor of NTCP with potent anti-HBV efficacy. **a** Identification of rapavir. Left-to-right: Construction of the rapafucin 3D microarrays and all of the compounds were spotted in duplicate. Chemical structures of HP07-C6 and JH-B10 (rapavir). Dose-dependent inhibition of TCA-d_4_ uptake, anti-HBV activity against HBeAg and HBV 3.5-kb RNA in HepG2-NTCP cells by HP07-C6 and rapavir. Data represent the mean ± s.d.; *n* = 3 samples. **b** Rapavir interacts with NTCP with relatively high specificity and its activity is independent of FKBP12 protein. Left-to-right: Chemical structure of biotin-rapavir. Affinity pulldown of detergent-solubilized NTCP by a biotin-rapavir conjugate and competition by free rapavir. Volcano plot analysis reveals a significant reduction in the levels of conjugated bile acids after treatment of rapavir for 6 h. FK506 did not antagonize rapavir inhibition of NTCP-mediated TCA-d_4_ uptake in HepG2-NTCP cells, and knockout of FKBP12 did not confer resistance to rapavir in HepG2-NTCP cells. **c** Rapavir blocks the HBV infection phase in HepG2-NTCP cells. Left-to-right: The intracellular HBV cccDNA levels were detected by Taq-man qPCR. The HBV 3.5-kb RNA levels were analyzed by RT-qPCR. HBeAg and HBsAg levels in the culture supernatant were detected by ELISA. Data represent the mean ± s.d.; *n* = 3 samples. **d** Rapavir resists HBV infection in humanized liver-chimeric mice. Left-to-right: HBV DNA in serum was measured by qPCR. Serum HBeAg was quantified by chemiluminescent immunoassay. HBV cccDNA and HBV 3.5-kb RNA in the liver tissues were determined by qPCR

To characterize the interaction between rapavir and NTCP, we synthesized a biotin affinity probe of rapavir (Fig. 1b). The biotin-rapavir conjugate was capable of pulling down NTCP and binding of NTCP to the biotin-rapavir probe is competed by free rapavir in a dose-dependent manner (Fig. 1b). This result further supported that rapavir directly interacts with NTCP. To explore the specificity of rapavir and understand the metabolic consequences of NTCP inhibition by rapavir, we measured the steady-state levels of 507 metabolites in HepG2-NTCP cells by LC–MS after 6 h of rapavir treatment. Metabolic analysis revealed that the most prominent metabolic alterations induced by rapavir were associated with conjugated bile acids instead of non-conjugates, which is consistent with the function of NTCP (Fig. 1b).^4^ This result suggested that rapavir has relatively high specificity for NTCP. Since rapavir contains a binding domain for FKBP (FKBD) like rapamycin and FK506, we next assessed whether inhibition of NTCP by rapavir is dependent on FKBP12 protein. A key characteristic of FKBP12 dependency is that the cellular effects can be counteracted by other FKBP12-binding ligands due to competition for FKBP12 protein. However, a high concentration of FK506 did not obviously affect the inhibitory potency of rapavir in the TCA-d_4_ uptake assay (Fig. 1b). The inhibitory activity of rapavir was also minimally affected by the knockout of FKBP12 (Fig. 1b). These findings indicated that the inhibitory effect of rapavir is FKBP12-independent.

To further confirm the role of rapavir in inhibiting the function of NTCP and its effect on the HBV infection phase, HepG2-NTCP cells were treated with rapavir either before or after inoculation with HBV particles. As expected, pretreatment with rapavir significantly reduced intracellular HBV cccDNA and HBV RNA, along with extracellular HBeAg and HBsAg, in a concentration-dependent manner (Fig. 1c). Conversely, the protective effect of rapavir on HepG2-NTCP cells was nearly abolished when it was added post-HBV inoculation (Fig. 1c). These results suggested that rapavir exerts its antiviral effect during the HBV infection phase by directly blocking NTCP, rather than impacting the viral replication process.

Having demonstrated the robust anti-HBV activity of rapavir in vitro, we next used the human liver-chimeric uPA/SCID mice to evaluate the antiviral efficacy of rapavir in vivo. The mice were allocated into two groups randomly. One group received intraperitoneal (i.p.) administration of rapavir (2 mg/kg body weight) 30 min before HBV infection. This was followed by repeated intraperitoneal administration 24 h after HBV infection, as well as on the 2nd, 3rd, and 5th days.^5^ While the control group of mice only received vehicle administration at the same time points. The experiment lasted for 8 weeks. As shown in Fig. 1d, the levels of serum HBV DNA and HBeAg persisted in rising following viral infection and rapidly increased from the 6th week to the 8th week in the control group. In contrast, the initial rise of serum HBV DNA and HBeAg was considerably prolonged, with relatively low levels detected even at the 8th week in the rapavir treatment group (Fig. 1d). Eight weeks following the viral infection, liver samples were harvested after the mice were euthanized. From these samples, we observed that the levels of intrahepatic HBV cccDNA and HBV RNA were remarkably lower in the group treated with rapavir than in the control group receiving the vehicle (Fig. 1d). Additionally, we assessed the hepatotoxicity and nephrotoxicity of rapavir and found that rapavir at doses up to 16 mg/kg did not exhibit significant toxicity in mice (data not shown). These results strongly suggested that rapavir was able to provide effective protection for liver cells against HBV infection in vivo without obvious side effects.

In summary, we discovered rapavir as a novel NTCP inhibitor from our rapafucin library via 3D microarray screening and SAR study. Rapavir was found to potently inhibit NTCP with relatively high specificity. It was also efficacious in blocking HBV infection in a human liver-chimeric mouse model in vivo without obvious side effects. Rapavir is one of the most potent NTCP inhibitors and the first small molecule that has been demonstrated to be efficacious in the human liver-chimeric mouse model, rendering it a new chemical tool for NTCP function studies and a potential candidate for the development of anti-HBV drugs.

## Supplementary information

Supplementary Material

## Data Availability

All the data used for the current study are available from the corresponding author upon reasonable request.
