# Supplementary material for: Rapavir, a novel inhibitor of sodium taurocholate cotransporting polypeptide, potently blocks hepatitis B virus entry
Source: Signal Transduct Target Ther. 2025 Apr 23;10:130. doi: 10.1038/s41392-025-02214-x (PMC12015363; doi:10.1038/s41392-025-02214-x)
Supplement: Supplementary file 1 — Supplementary Material [file 41392_2025_2214_MOESM1_ESM.docx]

Supplementary Materials for

Rapavir, a novel inhibitor of sodium taurocholate cotransporting polypeptide, potently blocks hepatitis B virus entry

Jia He^1†^, Haibo Yu^2†^, Kunling Song^1†^, Ailong Huang^2*^, Yongjun Dang^1*^, Juan Chen^2,3*^, Zufeng Guo^1*^

Correspondence to: ahuang@cqmu.edu.cn (A.H.); yjdang@cqmu.edu.cn (Y.D.); chenjuan2014@cqmu.edu.cn (J.C.); zfguo@cqmu.edu.cn (Z.G.)

**This PDF file includes:**

Materials and Methods

Materials and Methods

General reagents for chemistry

All reagents such as piperidine, N, N-d iisopropylethylamine (DIPEA), HATU, TBAF (1 M in THF) and anhydrous solvents were purchased from Adamas, Aladin, Accela, or Acros. Solid support resin with 2-chlorotrityl chloride and Fmoc protected amino acid building blocks were purchased from GL Biochem (Shanghai) Ltd. Iodoacetyl-PEG2-biotin was purchased from Thermo Fisher. Hoveyda-Grubbs catalyst 2^nd^ generation was purchased from Sigma-Aldrich.

Instruments for synthesis, purification, and detection

NMR spectra were recorded with Bruker 600. HPLC analyses were performed with Agilent 1290 Infinity II. Microwave reactions were performed with Multiwave Pro with silicon carbide 24-well blocks from Anton Parr. The high-resolution mass spectra (HRMS) were recorded on Thermo (Vanquish Flex+Orbit rap Exploris 120) and Agilent 7000D (ESI).

Synthesis of JH-B10 (Rapavir)

See references [1] for the general procedure. The crude product was purified by flash column chromatography (DCM: MeOH = 20: 1 to 10: 1) and high performance liquid chromatography (HPLC), affording 59.9 mg (31% in 11 steps) of pure JH-B10 as a pale yellow solid. ^1^H NMR (600 MHz, CDCl_3_) δ 7.26-7.05 (m, 8H), 7.02-6.74 (m, 8H), 6.73-6.57 (m, 6H), 5.86-5.62 (m, 2H), 5.35-5.24 (m, 1H), 5.12-4.87 (m, 2H), 4.78-4.54 (m, 2H), 4.50-4.39 (m, 1H), 4.36-4.14 (m, 3H), 3.86-3.82 (m, 6H), 3.60-3.38 (m, 2H), 3.34-3.16 (m, 2H), 3.06-2.94 (m, 5H), 2.92-2.69 (m, 5H), 2.68-2.46 (m, 6H), 2.43-2.17 (m, 5H), 2.15-2.00 (m, 3H), 1.79-1.59 (m, 4H), 1.56-1.44 (m, 2H), 1.36-1.29 (m, 3H), 1.28-1.25 (m, 3H) ppm; ^13^C NMR (150 MHz, CDCl_3_) δ 165.37, 161.09, 158.22, 155.65, 155.51, 152.07, 152.04, 148.90, 147.36, 145.90, 141.51, 133.44, 133.43, 133.35, 133.33, 132.54, 131.03, 131.01, 130.91, 130.85, 130.25, 130.23, 130.20, 130.05, 130.04, 129.91, 129.49, 129.09, 128.50, 127.07, 127.05, 124.05, 123.51, 122.71, 120.24, 120.20, 115.92, 115.81, 115.59, 115.48, 115.43, 111.90, 111.79, 111.38, 55.96, 55.89, 51.35, 50.03, 46.64, 44.11, 44.03, 37.41, 35.56, 34.01, 31.65, 31.31, 29.72, 29.69, 29.38, 29.35, 29.34, 22.48, 21.67, 21.55, 21.08, 21.02, 14.22 ppm. HRMS-ESI (m/z) calculated for [C_68_H_79_FN_6_O]^+^, 1239.5660; found, 1239.5631. HPLC: t_R_ = 2.091 min, purity: 96.6%.

Synthesis of biotin-Rapavir

See references [1] for the general procedure. The crude probe was purified by flash column chromatography (DCM: MeOH = 40: 1 to 20: 1) and afforded 3.0 mg (21% in two steps) biotin-rapavir. HRMS-ESI (m/z) calculated for [C_89_H_118_FN_10_O_20_S_3_]^+^, 1761.7665; found, 1761.7649.

General biological reagents

Dulbecco’s modified Eagle’s medium (DMEM) media were purchased from ThermoFisher Scientific (Cat#: C11995500BT). Taurocholic acid-d_4_ (TCA-d_4_) was purchased from Cayman (Cat#: 21891). Pierce™ Streptavidin magnetic beads was purchased from ThermoFisher Scientific (Cat#: 88816). Protein assay kit was purchased from Bioss (Cat#: C05-02001). Anti-his-tag and anti-FKBP12 antibodies were purchased from Cell Signaling Technology (Cat#: 12698) and selleck (Cat#: A5897), respectively.

Cell culture

HEK293T cells were obtained from the American Type Culture Collection (ATCC). HepAD38 cells were kindly provided by Prof. Ningshao Xia (Xiamen University, China). HepG2-NTCP stable cell line was established in our laboratory (J.C.).^2^ All cell lines were cultured in DMEM supplemented with 10% fetal bovine serum, 100 IU/mL penicillin, and 100 μg/mL streptomycin. All cell lines were incubated in a humidified incubator at 37°C with 5% CO_2_.

TCA-d_4_ uptake assay

See references [3] for the general procedure.

HBV preparation and infection

To prepare the HBV particles for infection, the culture supernatant of HepAD38 cells was collected. The collected supernatant was mixed with 6% PEG8000 and then concentrated by centrifugation at 4,000 × g for 30 minutes. The precipitate containing viral particles was dissolved in Opti-MEM (Gibco, USA) at a 100-fold concentration and the HBV genome equivalent was quantified by measuring HBV DNA with the absolute quantitative PCR assay. In the infection experiments, HepG2-NTCP cells were pretreated with compounds for 1 hour and then infected with HBV at 500 vge/cell in the presence of compounds and 4% w/v PEG 8,000 for 24 hours. Following the infection, the cells were washed and the culture medium was replaced every day until day 6. Then, the culture supernatants were collected to measure the levels of extracellular HBeAg and HBsAg, and the cells were harvested to quantify the levels of intracellular HBV RNA, HBV DNA, and HBV cccDNA.

Enzyme-linked immunosorbent assay (ELISA)

The culture supernatants were centrifuged at 2,000 g for 5 min, and the HBeAg and HBsAg in the supernatants were examined by enzyme-linked immunosorbent assay kit (Kehua, China) according to the manufacturer’s instructions.

RNA extraction and Real-time reverse-transcription PCR

Total cellular RNA was extracted by using TRNzol Universal reagent (DP424, TIANGEN, China), and the cDNA was synthesized from 1 μg extracted total RNA using an FastKing cDNA Synthesis Kit (With gDNase) (KR116, TIANGEN, China) according to the manufacturer’s manufacturer. Quantitative PCR (qPCR) was then conducted using the Universal SYBR Green Supermix (Bio-Rad, USA) with β-actin mRNA as an internal control. The fold change of HBV RNAs was calculated using the 2^-ΔΔCt^ method, which compared the threshold cycle (Ct) values of the target gene (HBV RNAs) to the reference gene (β-actin mRNA). The following primer sequences were used: HBV 3.5-kb RNA: Forward primer (s): GCCTTAGAGTCTCCTGAGCA, Reverse primer (as): GAGGGAGTTCTTCTTC TAGG; β-actin mRNA: Forward primer (s): CTCTTCCAGCCTTCCTTCCT, Reverse primer (as): AGCACTGTGTTGGCGTACAG.

Affinity pull-down assay with biotinylated rapavir

See references [1] for the general procedure. Briefly, HEK293T NTCP overexpression cells were washed once in buffer A and then incubated with buffer B at 4°C for 1 hour with constant mixing to extract NTCP protein. The supernatant was collected by centrifugation and then diluted in buffer A to reduce the concentration of DDM. The diluted supernatant was pre-incubated with streptavidin agarose beads at 4°C for 30 min. For a typical biotin affinity pull-down reaction, 300 µL of supernatant was pretreated with free rapavir or equal volume of DMSO for 30 min, before the addition of rapavir-biotin probe or DMSO. After incubation at 4°C for 1 h with constant mixing, 25 µL of streptavidin agarose beads in buffer A was added, and incubation was continued for 2 h. The agarose beads were washed three times with 0.5 mL of buffer A. The washed streptavidin agarose beads were then resuspended in 50 µL of 1 x SDS sample buffer, heated at 100°C for 5 min and was subjected to SDS-PAGE followed by western blot.

Metabolite extraction and analysis

HepG2-NTCP Cells were seeded into 10 cm dishes at a density of 3×10^6^ cells/well, allowed to recover overnight and subsequently treated with 100 nM rapavir or DMSO for 6 h. Metabolites were extracted and analyzed by the Shanghai bioprofile biotechnology company.

Determination of serum HBeAg and HBsAg

The serum HBeAg and HBsAg levels were quantified by using the Abbott Architect System with HBeAg or HBsAg Reagent Kit (Abbott, USA) according to the manufacturer’s instructions.

*In vivo* inhibitory effect of rapavir in chimeric mice

Human liver-chimeric uPA/SCID mice were generated by Beijing Vitalstar Biotechnology Co., Ltd. (Beijing, China) and housed under pathogen free conditions. Ten male mice (16 weeks of age) were randomly divided into experimental group and control group, with five mice in each. Intraperitoneal injection of 2 mg/kg rapavir or vehicle (5% DMSO in 0.5% methylcellulose) was performed during the HBV infection phase, and all the mice were inoculated with 10^6^ genotype C HBV genome equivalents by tail vein injection. Serum was collected from the orbital sinus every week. The mice were sacrificed at day 56, and liver tissues were collected for further study.

Statistical analysis

The results were expressed as the mean±SD. Statistical analyses were performed using either Student’s *t*-test or the Mann-Whitney U test. Differences were considered significant when *P*<0.05. All statistical analysis was performed by using GraphPad Prism software (GraphPad Software Inc., San Diego, CA).

**References**

[1] Guo, Z. et al. Rapamycin-inspired macrocycles with new target specificity. *Nat Chem.* **11**, 254-263 (2019).

[2] Cheng, S. T., et al. Dicoumarol, an NQO1 inhibitor, blocks cccDNA transcription by promoting degradation of HBx. *J Hepatol.* **74**, 522-534 (2021).

[3] Song, K. et al. A rapid and simple non-radioactive assay for measuring uptake by solute carrier transporters. *Front Pharmacol*. **15**, 13555507 (2024).
